# Exenatide compared with long-acting insulin to achieve glycaemic control with minimal weight gain in patients with type 2 diabetes: results of the Helping Evaluate Exenatide in patients with diabetes compared with Long-Acting insulin (HEELA) study

**DOI:** 10.1111/j.1463-1326.2009.01154.x

**Published:** 2009-12

**Authors:** M J Davies, R Donnelly, A H Barnett, S Jones, C Nicolay, A Kilcoyne

**Affiliations:** 1 Department of Cardiovascular Sciences, University of LeicesterLeicester, UK; 2 School of Graduate-Entry Medicine and Health, University of NottinghamNottingham, UK; 3 Division of Medical Sciences, University of Birmingham and Heart of England NHS Foundation TrustBirmingham, Birmingham, UK; 4 The Academic Centre, James Cook University HospitalMiddlesbrough, UK; 5 Lilly Deutschland GmbH, European MedicalBad Homburg, Germany; 6 Eli Lilly & Company LtdBasingstoke, Hampshire, UK

**Keywords:** exenatide, glucagon-like peptide-1 (GLP-1) agonist, glycaemic control, insulin glargine, randomized controlled trial, type 2 diabetes, weight gain

## Abstract

**Aim**: The Helping Evaluate Exenatide in overweight patients with diabetes compared with Long-Acting insulin (HEELA) study was designed to examine whether the glucagon-like peptide-1 (GLP-1) receptor agonist, exenatide, could improve HbA1c (≤7.4%) with minimal weight gain (≤1 kg) compared with insulin glargine.

**Methods**: Patients [body mass index (BMI) >27 kg/m^2^] with elevated cardiovascular risk and type 2 diabetes inadequately controlled on two or three oral antidiabetes drugs (OADs) were randomized to add-on exenatide 5–10 μg b.i.d. (n = 118) or insulin glargine o.d. (titrated to target fasting plasma glucose ≤5.6 mmol/l; n = 117) for 26 weeks.

**Results**: The study population had baseline mean (s.d.) age of 56.5 (9.1) years and BMI of 34.1 (5.3) kg/m^2^, and 58.5% of patients were taking two OADs. Mean baseline HbA1c was 8.65 (0.68)% in the exenatide group and 8.48 (0.66)% in the insulin glargine group. The proportions of patients achieving the composite endpoint of HbA1c ≤7.4% with weight gain ≤1 kg were 53.4% for the exenatide group and 19.8% for the insulin glargine group (p < 0.001 for exenatide vs. insulin glargine). Exenatide and insulin glargine did not demonstrate a significant difference in HbA1c improvements [least square (LS) mean [s.e.m.]: −1.25 [0.09]% and −1.26 [0.09]% respectively; p = 0.924], but had divergent effects on body weight (−2.73 [0.31] vs. +2.98 [0.31] kg respectively, p < 0.001) after 26 weeks. There were more treatment-related adverse events with exenatide but a lower incidence of nocturnal hypoglycaemia, with no differences in overall or severe hypoglycaemia.

**Conclusions**: Additional treatment with exenatide resulted in significantly more overweight and obese patients with an elevated cardiovascular risk and type 2 diabetes achieving better glycaemic control with minimal weight gain compared with insulin glargine.

## Introduction

Clinical management of overweight and obese patients with type 2 diabetes inadequately controlled despite two or more oral antidiabetes drugs (OADs) is particularly challenging [1]. This specific group of patients have a high risk of cardiovascular disease [2], and the ideal therapeutic goal is to improve glycaemic control with weight loss or minimal weight gain [3].

Use of combination OADs is often followed by insulin replacement, although these agents increase body weight to varying extents [4–6]. Use of basal insulin analogues, including both insulin glargine (Lantus) and insulin detemir (Levemir), in combination with OADs is associated with less weight gain than other insulin regimens [7–9].

Exenatide (Byetta) is a synthetic peptide that acts as an incretin mimetic and has been shown *in vitro* to bind to the glucagon-like peptide-1 (GLP-1) receptor [10]. It shares antidiabetes actions with the naturally occurring hormone GLP-1, including glucose-dependent enhancement of insulin secretion, suppression of inappropriately elevated glucagon levels, slowing of gastric emptying and reduction of food intake [11]. The effects of adding exenatide or insulin glargine to OAD therapy have been reported in several trials [12,13], in which exenatide was associated with a reduction in body weight [12], whereas insulin analogue therapy was associated with weight gain. Similar improvements in glycaemic control were achieved with either agent [13,14].

The Helping Evaluate Exenatide in patients with diabetes compared with Long-Acting insulin (HEELA) study was designed to compare treatment with exenatide vs. insulin glargine, on the composite primary endpoint of HbA1c ≤7.4% and weight gain ≤1 kg in a population of overweight patients with type 2 diabetes who were at high risk of cardiovascular disease and not adequately controlled by two or three OADs.

## Patients and Methods

### Study Population

This multicentre, randomized, open-label, parallel-arm, comparator study was undertaken in 36 centres in the UK between June 2006 and April 2008. Patients with type 2 diabetes [body mass index (BMI) >27 kg/m^2^] were eligible for the study if they had inadequate glycaemic control (HbA1c 7.5–10.0%), despite treatment with stable doses of two or three OADs (metformin, sulphonylurea and thiazolidinedione) for at least 3 months before randomization. Patients had at least one cardiovascular risk factor defined as either a previous cardiovascular event, peripheral vascular disease, or an abnormal risk factor [low-density lipoprotein (LDL) >3.0 mmol/l, high-density lipoprotein (HDL) <1.0 mmol/l (men) or <1.3 mmol/l (women), triglyceride >1.7 mmol/l, systolic blood pressure (BP) >130 mmHg, diastolic BP >80 mmHg or increased waist circumference (European: >94 cm, men, >80 cm, women; Asian: >90 cm, men, >80 cm, women)]. Exclusion criteria included history of malignancy, Class III or IV heart disease, uncontrolled hypertension (systolic BP ≥180 mmHg, diastolic BP ≥105 mmHg), renal transplantation or dialysis, chronic renal impairment (serum creatinine ≥135 μmol/l for males and ≥110 μmol/l for females) or liver disease (serum alanine aminotransferase >3 × upper limit of normal).

The study was performed following institutional ethical approval and according to the Declaration of Helsinki. All patients provided written informed consent.

### Study Design

Patients were randomized (in a 1 : 1 ratio) to exenatide or insulin glargine as add-on therapy for 26 weeks. Randomization was stratified according to the number (two or three) of OADs. All treatments were self-administered using reusable injection pens with prefilled cartridges. Exenatide was administered at 5 μg b.i.d. for the first 4 weeks, then 10 μg b.i.d. for the remainder of the study. Insulin glargine was initiated at 10 IU/day and titrated weekly according to a target fasting plasma glucose level ≤5.6 mmol/l (≤100 mg/dl). For mean self-monitored fasting plasma glucose levels ≥10 mmol/l, the increase in insulin glargine dosage was 8 IU/day; for fasting plasma glucose levels of 7.8–9.9 mmol/l, the increase in insulin glargine dosage was 6 IU/day and for fasting plasma glucose levels of 6.7–7.7 or 5.6–6.6 mmol/l, the increase in insulin glargine dosage was 4 or 2 IU/day respectively, as detailed previously [15]. Therapies were administered by subcutaneous injection in the abdomen, within 15 min before morning and evening meals for exenatide and once daily at bedtime for insulin glargine. Previous OADs were continued at the same stable dosages unless one or more confirmed or suspected hypoglycaemic event occurred, when the sulphonylurea dose could be reduced.

All patients attended clinic visits at screening, baseline and weeks 4, 8, 12, 18 and 26. Medical history, blood chemistry, haematology and physical examination, including height, weight, vital signs and electrocardiogram, were evaluated at screening. HbA1c concentrations were measured at screening, baseline and weeks 12 and 26; body weight was measured and safety assessed at each clinic visit. Laboratory measurements, including fasting serum lipids (total cholesterol, HDL cholesterol, triglycerides and LDL cholesterol), fasting serum glucose and clinical chemistry, were carried out at baseline and week 26.

All treatment emergent adverse events (TEAEs) were recorded and coded according to the Medical Dictionary for Regulatory Activities (MedDRA), version 9.0. Hypoglycaemic episodes were recorded and defined as incidents in which a patient experienced a sign or symptom associated with hypoglycaemia or who had a blood glucose <3.4 mmol/l (<60 mg/dl) even if it was not associated with a sign, symptom or treatment. Severe hypoglycaemia was defined as an episode with symptoms consistent with hypoglycaemia in which the patient required the assistance of a third party and also had an associated blood glucose level <2.8 mmol/l (50 mg/dl) and/or prompt recovery after oral carbohydrate, glucagon or intravenous glucose, and/or resulted in coma.

### Outcomes and Statistical Analysis

The primary efficacy variable was the proportion of patients with an HbA1c level ≤7.4% *and* weight gain ≤1 kg, at the end of the study. Although an HbA1c of 7.4% does not reflect optimal glycaemic control, this cut-off was chosen to reflect the UK audit standards for glycaemic control current at the time the study was performed [16]. The weight change of a gain ≤1 kg was a pragmatic choice that reflected the likely variation in sequential measurements of weight. The sample size of 234 patients was calculated to provide a power of 90% to detect a difference in the assumed primary response rate of 51% (exenatide) vs. 27% (insulin glargine), with a two-sided significance level of 5% and a discontinuation rate of 20%. Because the most frequently used target is currently HbA1c <7.0% [3,6], and the UK quality and outcome framework has recently been changed to reflect this, a *post hoc* analysis was carried out using a composite endpoint of HbA1c ≤7.0% with weight gain ≤1 kg to examine changes in relation to recent practice.

All analyses were performed on a modified intent-to-treat basis in randomized patients who received at least one dose of study drug [full analysis set (FAS)]. The primary efficacy analysis was based on a logistic regression model for the treatment group, with number of OADs (two or three) as a factor. The last postbaseline measurement set of both non-missing HbA1c and weight was used as the endpoint value. Patients with no baseline weight measurements and/or missing postbaseline measurements for HbA1c and/or weight were included as non-responders in the analysis. Categorical secondary measures were analysed accordingly.

Continuous secondary measures were explored using a mixed model repeated measurement (MMRM) analysis with independent variables being treatment group, visit, the interaction between treatment group and visit, and the corresponding baseline value. For continuous variables that were only measured twice, an analysis of covariance (ancova) model was used, with the change from baseline to endpoint as the dependent variable and treatment group, corresponding baseline value and number of OADs administered, as independent variables. Fisher's exact tests were used for the treatment group comparison of discontinuation and adverse event percentages.

All analyses were prespecified and carried out using sas version 9.1.3 (SAS Institute Inc., Cary, NC, USA). Tests of treatment effects were conducted at a two-sided significance level of 5% and all had 95% confidence intervals (CIs). The difference between treatments was calculated on the basis of exenatide vs. insulin glargine or exenatide minus insulin glargine, depending on the type of analysis.

## Results

### Patient Demographics and Characteristics at Baseline

**Fig. 1:**
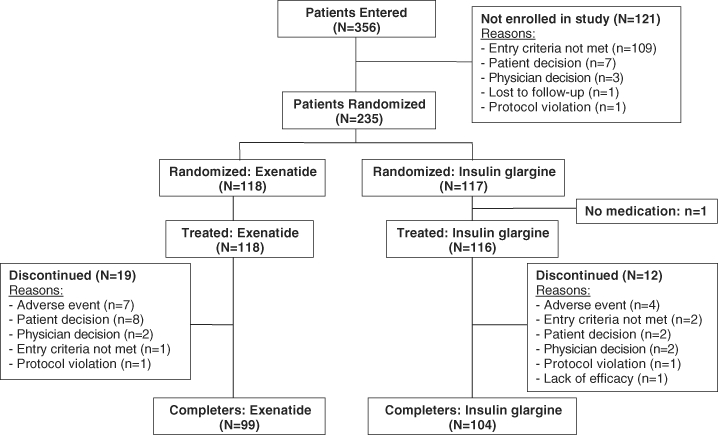
Flow diagram of subject disposition.

There were 118 patients randomized to exenatide (figure 1) and 117 patients randomized to insulin glargine (116 treated). In the exenatide group, 19 (16.1%) patients discontinued treatment, and in the insulin glargine group 12 (10.3%) discontinued (p = 0.248).

The proportions of patients taking two (58.5% overall) or three (40.6% overall) OADs and with pre-existing conditions (97.0% overall) at baseline were similar in both treatment groups (table 1). Most patients were taking a combination of metformin and sulphonylurea (42.3%) or metformin, sulphonylurea and thiazolidinedione (40.6%). The majority of patients in each treatment group were taking a sulphonylurea (exenatide 84.7% and insulin glargine 86.2%). Hypertension (70.1%) and dyslipidaemia (31.6%) were the most common concomitant conditions reported.

**Table 1:** Baseline characteristics of patients receiving exenatide or insulin glargine

Variable	Exenatide (n = 118)	Insulin glargine (n = 116)	Total (N = 234)
Males [n (%)]	83 (70.3)	77 (66.4)	160 (68.4)
Age [mean (s.d.)] (years)	56.8 (10.2)	56.2 (7.9)	56.5 (9.1)
Duration of diabetes [mean (s.d.)] (years)	9.0 (4.6)	8.4 (4.4)	8.7 (4.5)
Weight [mean (s.d.)] (kg)	101.4 (19.8)	97.6 (16.4)	99.5 (18.3)
Body mass index [mean (s.d.)] (kg/m^2^)	34.6 (5.7)	33.7 (4.9)	34.1 (5.3)
Waist/hip ratio [mean (s.d.)]	0.99 (0.08)	0.98 (0.08)	0.98 (0.08)
HbA1c [mean (s.d.)] (%)	8.65 (0.68)	8.48 (0.66)	8.57 (0.67)
Fasting serum glucose [mean (s.d.)] (mmol/l)	10.84 (2.23)	10.12 (2.22)	10.48 (2.25)
Systolic BP [mean (s.d.)] (mmHg)	134.0 (14.9)	134.5 (17.5)	134.2 (16.2)
Diastolic BP [mean (s.d.)] (mmHg)	79.5 (8.4)	79.8 (9.2)	79.7 (8.8)
Triglycerides [mean (s.d.)] (mmol/l)	1.94 (1.01)	2.31 (2.24)	2.13 (1.75)
Total cholesterol [mean (s.d.)] (mmol/l)	4.45 (1.05)	4.63 (1.20)	4.54 (1.13)
LDL [mean (s.d.)] (mmol/l)	2.38 (0.82)	2.49 (1.02)	2.43 (0.93)
HDL [mean (s.d.)] (mmol/l)	1.14 (0.24)	1.15 (0.29)	1.15 (0.27)
Type of oral diabetes agent* [n (%)]			
Metformin/sulphonylurea	50 (42.4)	49 (42.2)	99 (42.3)
Metformin/thiazolidinedione	17 (14.4)	15 (12.9)	32 (13.7)
Sulphonylurea/thiazolidinedione	2 (1.7)	4 (3.4)	6 (2.6)
Metformin/sulphonylurea/thiazolidinedione	48 (40.7)	47 (40.5)	95 (40.6)
Pre-existing conditions [n (%)]			
Hypertension	85 (72.0)	79 (68.1)	164 (70.1)
Dyslipidaemia	35 (29.7)	39 (33.6)	74 (31.6)
Hypercholesterolaemia	21 (17.8)	19 (16.4)	40 (17.1)
Microvascular complications [n (%)]	31 (26.3)	38 (32.8)	69 (29.5)
Macroangiopathy† [n (%)]	16 (13.6)	21 (18.1)	37 (15.8)

* Additional two patients only had one OAD at baseline and for analysis were included in the two OADs group.

† Macroangiopathy included angina pectoris, cerebral infarction, cerebrovascular accident, coronary artery disease, intermittent claudication, myocardial ischaemia, peripheral vascular disorder and transient ischaemic attack.

### Effect of Exenatide and Insulin Glargine on Glycaemic Indicators and Weight

**Fig. 2:**
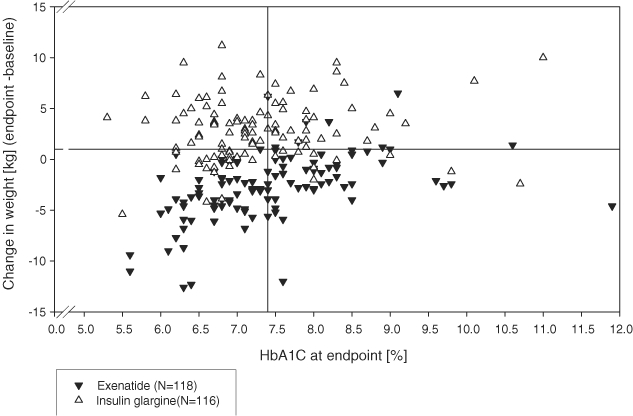
Scatter plot of changes in body weight vs. HbA1c concentration after 26 weeks of exenatide or insulin glargine treatment in patients with type 2 diabetes; the horizontal line shows a weight gain of 1 kg and the vertical line shows an HbA1c of 7.4%.

The change in weight is plotted against the endpoint HbA1c in figure 2. Compared with insulin glargine, exenatide was significantly more effective in achieving the primary composite endpoint of HbA1c reduction to ≤7.4% with a weight gain of ≤1 kg [odds ratio (OR): 4.71, 95% CI: 2.62–8.46, p < 0.001; table 2]. The number of patients achieving this composite endpoint after 26 weeks was 63 (53.4%) in the exenatide group vs. 23 (19.8%) in the insulin glargine group. In each treatment group, five patients were analysed as non-responders because of missing baseline and/or endpoint values for weight and/or HbA1c. The supportive primary analysis on patients with non-missing values showed similar results (OR: 4.87, 95% CI: 2.69–8.82, p < 0.001) to those obtained with the FAS. A *post hoc* analysis using an endpoint HbA1c of ≤7.0% with a weight gain of ≤1 kg showed that 46 (39.0%) patients in the exenatide group and 21 (18.1%) in the insulin glargine group achieved this composite endpoint (OR: 2.90, 95% CI: 1.59–5.28, p < 0.001). The median dose of insulin glargine at endpoint was 34.0 (interquartile range: 24.0–52.0) IU/day and the mean (s.d.) dose was 38.7 (23.5) IU/day.

**Table 2:** HbA1c, weight, fasting serum glucose, lipid and blood pressure values in patients receiving exenatide or insulin glargine after 26 weeks of treatment

Variable	Exenatide					Insulin glargine			Between treatment p value
HbA1c ≤7.4%, weight gain ≤1 kg	N				N				
n (%)	118	63 (53.4)		—	116	23 (19.8)		—	
[95% confidence interval (CI)]		[44.0, 62.6]				[13.0, 28.3]			
Odds ratio [95% CI]				4.71 [2.62, 8.46]					<0.001*
	**n**	**Actual measurement**	**n**	**Change from baseline**	**n**	**Actual measurement**	**n**	**Change from baseline**	
HbA1c (%)									
Least square (LS) mean (s.e.m.)*	98	7.32(0.09)	98	−1.25 (0.09)	102	7.31 (0.09)	102	−1.26 (0.09)	0.924†
[95% CI]		[7.14,7.50]		[−1.43, −1.07]¶		[7.13, 7.49]		[−1.44, −1.08]¶	
Weight (kg)									
LS mean (s.e.m.)*	100	97.12(0.31)	100	−2.73 (0.31)	104	102.83 (0.31)	104	+2.98 (0.31)	<0.001†
[95% CI]		[96.5,97.7]		[−3.34, −2.11]¶		[102.2, 103.4]		[2.37, 3.60]¶	
	**n**	**Actual measurement, mean (s.d.)**	**n**	**Change from baseline, LS mean (s.e.m.)**	**n**	**Actual measurement, mean (s.d.)**	**n**	**Change from baseline, LS mean (s.e.m.)**	
Waist circumference (cm)	117	110.2(14.5)	117	−1.90 (0.52)¶	114	112.6 (12.8)	114	1.86 (0.53)¶	<0.001‡
Fasting serum glucose (mmol/l)	105	8.51(2.55)	103	−2.12 (0.25)¶	104	6.78 (2.67)	101	−3.61 (0.25)¶	<0.001‡
Triglycerides (mmol/l)	108	1.72(0.92)	105	−0.33 (0.08)¶	108	1.79 (1.03)	108	−0.38 (0.08)¶	0.650‡
Total cholesterol (mmol/l)	108	4.09(0.95)	105	−0.36 (0.07)¶	108	4.36 (0.97)	108	−0.21 (0.07)§	0.125‡
LDL (mmol/l)	104	2.15(0.77)	95	−0.25 (0.06)¶	105	2.35 (0.84)	101	−0.07 (0.05)	0.017‡
HDL (mmol/l)	106	1.15(0.27)	101	0.01 (0.01)	106	1.17 (0.28)	106	0.02 (0.01)	0.471‡
Systolic blood pressure (BP) (mmHg)	117	131.5(15.2)	117	−2.9 (1.2)**	114	135.5 (17.2)	114	0.7 (1.2)	0.034‡
Diastolic BP (mmHg)	117	79.3(8.9)	117	−0.5 (0.7)	114	80.8 (8.7)	114	0.9 (0.7)	0.158‡

The n values show the number of patients with evaluable data for each variable examined.

* From logistic regression analysis.

† From mixed model repeated measurement analysis.

‡ From analysis of covariance.

¶ p < 0.001 for within-group change from baseline.

§ p = 0.004 for within-group change from baseline.

** p = 0.015 for within-group change from baseline.

Changes in HbA1c, weight, waist circumference, fasting blood glucose, lipid profile and BP are summarized in table 2. Decreases in LS mean values for HbA1c from baseline after 12 and 26 weeks of either treatment were statistically significant (p < 0.001 for all). The LS mean difference in the decrease in HbA1c between the exenatide and insulin glargine groups was significant after 12 weeks (−0.17%, 95% CI: −0.33 to 0.00%; p = 0.044), but not at 26 weeks (0.01%, 95% CI: −0.24 to +0.27%; p = 0.924).

**Fig. 3:**
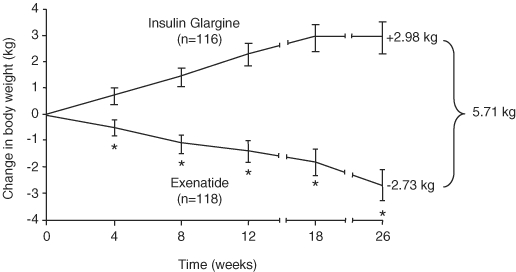
Changes in body weight of patients with type 2 diabetes during treatment with either exenatide or insulin glargine; values for each group are least square means with 95% confidence intervals from mixed model repeated measurement analysis. ^*^p < 0.001 exenatide vs. insulin glargine at the same time point.

Mean body weight decreased with exenatide but increased following insulin glargine treatment (figure 3). The LS mean difference between the changes from baseline in the two groups was significant at 4 weeks (−1.19 kg, 95% CI: −1.63 to −0.75 kg; p < 0.001) and all subsequent timepoints: 12 weeks (−3.74 kg, 95% CI: −4.34 to −3.14 kg, p < 0.001); 26 weeks (−5.71 kg, 95% CI: −6.58 to −4.84 kg, p < 0.001). Waist circumference was also statistically significantly decreased in the exenatide group and increased in the insulin glargine group (table 2), resulting in a significant endpoint difference between treatments (p < 0.001).

The mean [s.d.] decrease from baseline in fasting serum glucose at endpoint was greater with insulin glargine (−3.41 [2.85] mmol/l) than with exenatide (−2.29 [2.64] mmol/l), and the LS mean difference between the treatments (+1.49, 95% CI: 0.80 to 2.18 mmol/l) was statistically significant (p < 0.001). The proportions of patients who achieved fasting serum glucose ≤5.6 mmol/l were 10.2% with exenatide (n = 12) and 32.8% with insulin glargine (n = 38). At 26 weeks, there was a significant decrease from baseline in total cholesterol and triglyceride concentrations in both treatment groups, and for LDL cholesterol with exenatide but not with insulin glargine (table 2). There was a statistically significant decrease from baseline to endpoint in systolic BP with exenatide treatment, which was statistically significantly different from the change with insulin glargine (LS mean difference −3.6 mmHg; p = 0.034), but no statistically significant change in diastolic BP with exenatide or insulin glargine treatment.

### Tolerability

The number of patients with TEAEs (table 3) was 106 (89.8%) in the exenatide and 94 (81.0%) in the insulin glargine groups (*p* = 0.065). The number of patients with a TEAE considered possibly drug-related was statistically significantly greater with exenatide (n = 91, 77.1%) than with insulin glargine (n = 12, 10.3%; p < 0.001). Gastrointestinal events occurred more commonly in patients receiving exenatide (70.3%) than those receiving insulin glargine (21.6%), with nausea being the most common (48.3% in patients receiving exenatide compared with 2.6% in patients receiving insulin glargine). Seven patients in the exenatide group discontinued treatment because of adverse events of nausea (two patients), vomiting (two patients) and diarrhoea, acute renal failure and rash (one patient for each) and four patients discontinued in the insulin glargine group because of adverse events of injection site pain, asthma, dyspnoea and rash (one patient for each). A higher incidence of nervous system disorders with exenatide was seen, although this was largely attributable to a higher incidence of dysgeusia (8.5% of the exenatide group vs. 0% of the insulin glargine group).

**Table 3:** Treatment emergent adverse events (TEAEs) by MedDRA system organ class (SOC) and preferred term and incidence of hypoglycaemic episodes during exenatide and insulin glargine treatment for 26 weeks

	Exenatide (n = 118) [n (%)]	Insulin glargine (n = 116) [n (%)]	Total (N = 234) [n (%)]
Patients with at least 1 TEAE	106 (89.8)	94 (81.0)	200 (85.5)
MedDRA SOC			
Preferred term*			
Gastrointestinal disorders	83 (70.3)	25 (21.6)	108 (46.2)
Nausea	57 (48.3)	3 (2.6)	60 (25.6)
Diarrhoea	22 (18.6)	14 (12.1)	36 (15.4)
Infections and infestations	43 (36.4)	55 (47.4)	98 (41.9)
Nasopharyngitis	24 (20.3)	23 (19.8)	47 (20.1)
Nervous system disorders	36 (30.5)	23 (19.8)	59 (25.2)
Headache	17 (14.4)	18 (15.5)	35 (15.0)
Patients with at least one SAE	5 (4.2)	5 (4.3)	10 (4.3)
Patients with at least one SAE possibly related to study drug	1 (0.8)	0 (0.0)	1 (0.4)
Acute myocardial infarction	1 (0.8)	0 (0.0)	1 (0.4)
Supraventricular tachycardia	1 (0.8)	0 (0.0)	1 (0.4)
Discontinued because of TEAE	7 (5.9)	4 (3.4)	11 (4.7)
Hypoglycaemia incidence†			
All episodes			
n (%)	59 (50.0)	68 (59.6)	127 (54.7)
[95% confidence interval (CI)]	[40.7, 59.3]	[50.1, 68.7]	[48.1, 61.3]
Odds ratio (OR) [95% CI]	0.68, 95% CI: 0.40–1.14, p = 0.139		
Episodes confirmed by blood glucose <3.4 mmol/l			
n (%)	37 (31.4)	42 (36.8)	79 (34.1)
[95% CI]	[23.1, 40.5]	[28.0, 46.4]	[28.0, 40.5]
OR [95% CI]	0.78, 95% CI: 0.45–1.35, p = 0.369		
Nocturnal hypoglycaemia			
n (%)	14 (11.9)	34 (29.8)	48 (20.7)
[95% CI]	[6.6, 19.1]	[21.6, 39.1]	[15.7, 26.5]
OR [95% CI]	0.32, 95% CI: 0.16–0.63, p = 0.001		
Severe hypoglycaemia			
n (%)	5 (4.2)	6 (5.3)	11 (4.7)
[95% CI]	[1.4, 9.6]	[2.0, 11.1]	[2.4, 8.3]
OR [95% CI]	0.80, 95% CI: 0.24–2.71, p = 0.716		

* Events that occurred in >10% in either treatment group.

† Confidence intervals are based on the exact method, p values are from logistic regression analysis.

The numbers of patients who discontinued due to adverse events were not statistically significantly different between the two treatment groups (p = 0.539). Five patients in each group reported at least one serious adverse event (table 3).

### Hypoglycaemia

There was no significant difference between exenatide and insulin glargine in overall incidence of symptomatic hypoglycaemic episodes (p = 0.139) and hypoglycaemic episodes confirmed by blood glucose measurements <3.4 mmol/l (p = 0.369) (table 3). However, the incidence of nocturnal hypoglycaemic episodes was significantly lower in the exenatide group than in the insulin glargine group (p = 0.001).

## Discussion

In the HEELA study, the proportion of patients with a final HbA1c ≤7.4% and weight gain ≤1 kg was significantly higher in the exenatide than in the insulin glargine group. This conclusion was supported by a *post hoc* analysis showing that a significantly higher proportion of the exenatide group than the insulin glargine group achieved a composite endpoint of HbA1c ≤7.0% and weight gain ≤1 kg. A limitation of the present study was the open-label nature, which was necessary because of the different regimens of the study treatments; a double-dummy design would have enabled blinding but would have required a much larger number of injections for each patient. Significant reductions from baseline in HbA1c in these high-risk patients with type 2 diabetes, inadequately controlled with two or more OADs, were similar in both treatment groups, but patients with exenatide treatment had a significantly greater decrease in weight compared with those treated with insulin glargine, where there were increases in weight, over 26 weeks. Although the number of patients with a TEAE considered possibly drug-related was greater with exenatide, there was no statistically significant difference in TEAEs overall or withdrawals because of adverse events between exenatide and insulin glargine. The overall incidence of hypoglycaemic episodes, including severe episodes, was not significantly different between the two groups, although it should be noted that the majority of patients in each group were receiving sulphonylureas. The incidence of nocturnal hypoglycaemic episodes was significantly less in the exenatide group.

The overweight and obese patients failing on two or three OADs in the present study were representative of a problematic group encountered in clinical practice. For such patients whose HbA1c levels remain above the target value, despite treatment with OADs, decisions on the next step of additional glucose-lowering therapy often represents a dilemma [17]. Where two OADs are administered, the addition of a third agent, such as a thiazolidinedione, is an option. However, such treatment results in weight gain and may be associated with undesirable side-effects of oedema, congestive heart failure and, for women, increased fracture risk [4,18]. Therefore, basal insulin is often implemented as add-on therapy [15,19], and the efficacy of insulin glargine was confirmed in the present study in a patient population with long duration of disease, who were overweight and had suboptimal glycaemic control despite combination OADs.

Obesity is an independent risk factor for cardiovascular disease and mortality in patients with type 2 diabetes [20]. In the present study, treatment with exenatide resulted in weight reduction. Other head-to-head studies of exenatide vs. insulin regimens showed similar significant weight differences in favour of exenatide [13,21]. In addition, in the present study, there were reductions with exenatide in waist circumference, triglycerides, total cholesterol, LDL cholesterol and systolic BP, with no effect on diastolic BP.

Reductions in HbA1c levels and weight changes in patients treated with exenatide or insulin glargine in the present study were similar to those observed in a crossover trial over 16 weeks [12] and in the Heine *et al*. [13] open-label study over 26 weeks. Reservations have been expressed with regard to the latter study because the final mean dose of insulin glargine (25 IU/day) was relatively low, even though it was titrated to maintain fasting plasma glucose <5.6 mmol/l. The lower dose in that study [13] may be partly explained by lower baseline HbA1c and BMI than for patients in the present study where the final mean insulin glargine dose (38.7 IU/day) was comparable with other studies [18,22]. In the present study, the interquartile range for insulin glargine dose at endpoint was 24–52 IU/day, indicating that at least 25% of the patients were receiving more than 50 IU/day. Thus, in our study, more robust comparative data on the use of exenatide or insulin glargine has been provided. Although the mean fasting glucose achieved with insulin glargine in the present study was 6.78 mmol/l, and only 32.6% of the patients achieved the target of ≤5.6 mmol/l, the study was only 26 weeks, which may have been insufficient to fully control fasting glucose in those patients who had high baseline values.

The importance of glucose control in reducing the complications of type 2 diabetes is well established. However, in two recent large studies of high-risk patients with type 2 diabetes, ADVANCE (Action in Diabetes and Vascular Disease: Preterax and Diamicron Modified Release Controlled Evaluation) [23] and ACCORD (Action to Control Cardiovascular Risk in Diabetes) [24], intensive treatment to achieve near-normal HbA1c levels (6%) did not significantly reduce cardiovascular events compared with standard targets (7.0–7.9%). In the ACCORD trial, rapid and aggressive control of glucose was associated with significantly increased risk of death from cardiovascular disease, although this finding was not supported by the ADVANCE study. The factors that may have influenced these different outcomes [25] are relevant in terms of the outcomes of the present study. In the ADVANCE trial, there was little change in body weight, whereas in the ACCORD trial a subgroup of patients gained substantial weight, with 28% gaining more than 10 kg and 27% of patients reported severe hypoglycaemia. In the present study, exenatide improved HbA1c levels but did not result in increase in weight and the frequency of nocturnal hypoglycaemia was reduced. Thus, exenatide may have a role in treating individuals at high risk of cardiovascular disease although long-term outcome studies are required.

The increased occurrence of gastrointestinal adverse events, such as nausea and diarrhoea, with exenatide compared with insulin glargine may limit its use. However, it is reassuring that, although gastrointestinal side-effects are common, few patients discontinued as a result of such side-effects.

In conclusion, the present study provides useful data on alternative therapies for patients with type 2 diabetes and high risk of cardiovascular disease that are often encountered in clinical practice. Further studies are required to assess the potential of these different agents in the longer-term treatment of type 2 diabetes.
